# Indications of chemical bond contrast in AFM images of a hydrogen-terminated silicon surface

**DOI:** 10.1038/ncomms14222

**Published:** 2017-02-13

**Authors:** Hatem Labidi, Mohammad Koleini, Taleana Huff, Mark Salomons, Martin Cloutier, Jason Pitters, Robert A. Wolkow

**Affiliations:** 1 Department of Physics, University of Alberta, Edmonton, Alberta, Canada T6G 2J1; 2 National Institute for Nanotechnology, National Research Council of Canada, Edmonton, Alberta, Canada T6G 2M9

## Abstract

The origin of bond-resolved atomic force microscope images remains controversial. Moreover, most work to date has involved planar, conjugated hydrocarbon molecules on a metal substrate thereby limiting knowledge of the generality of findings made about the imaging mechanism. Here we report the study of a very different sample; a hydrogen-terminated silicon surface. A procedure to obtain a passivated hydrogen-functionalized tip is defined and evolution of atomic force microscopy images at different tip elevations are shown. At relatively large tip-sample distances, the topmost atoms appear as distinct protrusions. However, on decreasing the tip-sample distance, features consistent with the silicon covalent bonds of the surface emerge. Using a density functional tight-binding-based method to simulate atomic force microscopy images, we reproduce the experimental results. The role of the tip flexibility and the nature of bonds and false bond-like features are discussed.

## Abstract (web summary)

Whether and under what circumstances chemical bonds could be imaged via force microscopy is a controversial topic. Here authors develop a particular combination of model surface, imaging procedures and simulation approach and discuss possible indications of chemical contrast in imaging data they obtain.

Since the pioneering work of Gross *et al*.^1^, many studies have reported submolecular resolution atomic force microscopy (AFM) images of different molecules and molecular assemblies revealing a chemical bond contrast^2^,^3^,^4^,^5^,^6^,^7^,^8^,^9^,^10^. Observing such contrast usually requires the use of a qPlus sensor^11^ at liquid helium temperature to achieve: (i) high stability and low signal to noise, (ii) small tip-sample distances and (iii) the controlled functionalization of the tip apex, usually with a CO molecule. Although very recently, a few studies showed liquid nitrogen^12^ and room temperature^13^,^14^ submolecular resolution.

Despite these experimental achievements and related theoretical studies, the origin and predominant factors to obtain such contrast are still a hot topic of debate in the AFM community^15^,^16^,^17^. The chemical bond contrast observed is usually interpreted as either the intramolecular structure of molecules or intermolecular bonds. Initially, it was suggested that this contrast arises from the Pauli repulsive force that becomes dominant at small tip-sample distances^1^,^18^. Later, based on a classical force field model^9^,^19^, the flexibility of the tip was claimed to be the dominant effect leading to the chemical bond contrast in AFM images. Also, a controversy arose following the claimed intermolecular hydrogen bond imaging by Zhong *et al*.^20^ that some subsequent studies ascribed to an artifact due to the CO tip flexibility and not necessarily hydrogen bonds^9^,^10^,^21^. Recently, Guo *et al*.^22^ showed that bond-like features can appear between bonded and non-bonded atoms due to the overlapping of the outermost electrons. Their density functional theory (DFT)-based approach which does not account for Pauli repulsion from electron density overlapping between the tip and sample, showed that the contrast in the NC-AFM images originates from the short-range electrostatic force^22^,^23^. Moreover, the CO tip flexibility was shown to enhance the bond-like contrast but is not necessary to observe it. This was confirmed in a more recent study by Monig *et al*.^17^ where a bond-like contrast could be seen experimentally and reproduced theoretically using a rigid Cu tip with an oxygen apex.

So far, this debate about the origin and mechanism of the bond-like contrast in AFM images focused almost exclusively on the specific case of molecules adsorbed on metal surfaces and imaged using a functionalized CO tip. In this article, we extend the parameter space and report what appears to possibly correspond to the observation of chemical bond contrast when imaging the H-terminated Si(100) surface revealing a feature consistent with the silicon covalent bond structure of the 2 × 1 reconstruction. This non-planar surface exhibits various Si–Si and Si–H bonds at different orientations with the H atoms near perpendicular to the surface plane. The dimer units present features unlike those probed in any previous AFM study. First, the Si–Si dimer is parallel to the surface plane, providing the first non-adsorbed molecule subject to test the AFM's ability to image bonds. The bond is also uncommonly long, ∼2.4 Å compared with <1.5 Å for the carbonaceous species studied to date, allowing the probe greater access to the space between covalently bonded atoms. Termination of the dimer with H atoms forces the constituent silicon atoms to retain an *sp*^3^-like character, thereby preventing the dimer from buckling substantially out of the surface plane^24^. Another unique feature of the dimer as a specimen for AFM study is its purely *σ* bond character. Unlike the *π* bonds studied to date, the *σ* bond decays more sharply in the direction perpendicular to the surface and therefore more nearly approximates the ‘stick bond' we schematically draw between covalently bonded atoms. Lastly, the fixed and exactly known proximity of 2 H atoms on two H-terminated dimers aligned end to end (that is two dimers in adjacent dimer rows) provides a wonderful opportunity to test recent conjectures related to false indications of bonds when H-containing molecules are closely juxtaposed.

One remaining challenge for NC-AFM experiments on semiconductors in general is preparing and identifying a stable tip capable of producing chemical bond contrast. Here we show that following *ex situ* cleaning with ebeam and field ion microscopy (FIM), a qPlus senor with a tungsten tip can be prepared *in situ* with the hydrogen-terminated silicon surface to obtain either a reactive or a passivated tip, both identified from the typical force curves they generate. Using a hydrogen-passivated tip, we study the evolution of the frequency shift maps contrast as a function of tip-sample distance. We show that for small tip-sample distances, the AFM images change from atom-like to chemical bond-like contrast.

We used a density functional tight-binding (DFTB)-based approach to efficiently simulate AFM images at precisions on par with DFT. We discuss the observed contrast at different tip-elevations as well as the role of the tip flexibility in the imaging mechanism.

## Results

### Making and identifying a hydrogen-passivated tip

**Figure 1:**
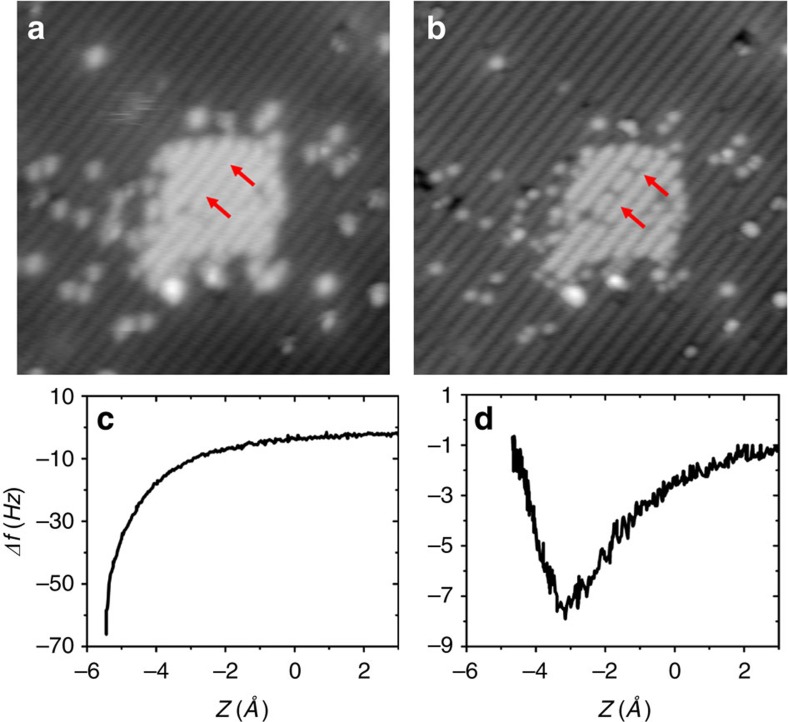
Making and identifying a hydrogen-passivated tip. (**a**) (20 × 20) nm^2^ constant current (30 pA, −2.0 V) STM image of the H–Si surface with a (5 × 5) nm^2^ bare silicon area appearing as a bright square at the centre of the image, and obtained with tip-induced hydrogen desorption. Following tip shaping procedure, the STM image becomes very sharp (**b**) and no longer shows the double tip effect visible in **a**. Red arrows indicate the location of the tip-induced silicon dimer hydrogen termination. (**c**,**d**) Frequency shift versus tip-sample distance of a reactive and a passivated tip, respectively.

Thanks to the *ex situ* tip cleaning procedure using ebeam and FIM (see experimental methods section for details), we always get scanning tunnelling microscopy (STM) atomic resolution of the surface right after the approach. However, images often exhibit artifacts, such as a double/multiple tip as seen in Fig. 1a, that renders data interpretation inaccurate. Therefore, it is necessary to further process the tip by *in situ* techniques to obtain a single atom tip apex. When studying metal surfaces, this is usually done by applying large voltage pulses and harsh indentation of the tip into the surface, followed by functionalizing the tip using a molecule such as CO^1^. Unfortunately, intentional functionalization of tips when studying semiconductor surfaces has not been achieved so far. Hence, one must rely on repeating controlled crashes and voltage pulses until the tip yields STM images of the surface with no artifacts, which reflects a single atom tip. Since the tip in our experiments was already cleaned from its oxide layer in the FIM, there is no need for us to apply high-voltage pulses and harsh-controlled crashes as previously described in AFM studies of silicon surfaces^25^,^26^. Instead, we use a more gentle procedure that gives stable tips without ravaging the studied surface area.

We start by bringing the tip in controlled contact with the silicon surface, which produces a silicon tip apex^26^. A bare silicon area is then created on the H–Si(100) surface by tip-induced hydrogen desorbtion^27^,^28^,^29^. In the example of Fig. 1a, a (5 × 5) nm^2^ square area is created by scanning at 4 V and 150 pA for about 6 min, coating the silicon tip apex with hydrogen. The tip is then brought close to the bare silicon area before a new STM image is acquired to check possible tip changes. This procedure is repeated until a sharp artifact-free STM image of the surface is obtained as shown in Fig. 1b. The small dark features indicated by red arrows in Fig. 1b are H-terminated silicon dimers created after the above tip preparation and H termination process was complete. The tip was positioned over a hydrogen-free dimer, then moved ∼6 Å closer to the surface. Re imaging revealed the newly H-terminated dimer. Similarly prepared H-terminated dimers have been previously described^30^. This capping of silicon dimers with H atoms was done to indicate the presence of multiple H atoms on the tip as a result of the H desorption preparation process. Ordinarily, however, we do not purposefully remove H atoms in this way.

The passivated character of the tip was further confirmed using force spectroscopy. Typical force curves of the H–Si surface acquired before the hydrogen desorption procedure, as in the example of Fig. 1c, clearly show a very reactive character. On the other hand, force curves acquired afterwards, as in Fig. 1d, show a passivated character. This is reminiscent of the difference observed between reactive metal tips and CO-passivated tips^23^,^31^. We also note here that approaching the H-passivated tip very close to the surface can result in changing the tip back to reactive. Therefore, it is possible to switch between reactive and passivated tips.

### Resolving atomistic contrast

**Figure 2:**
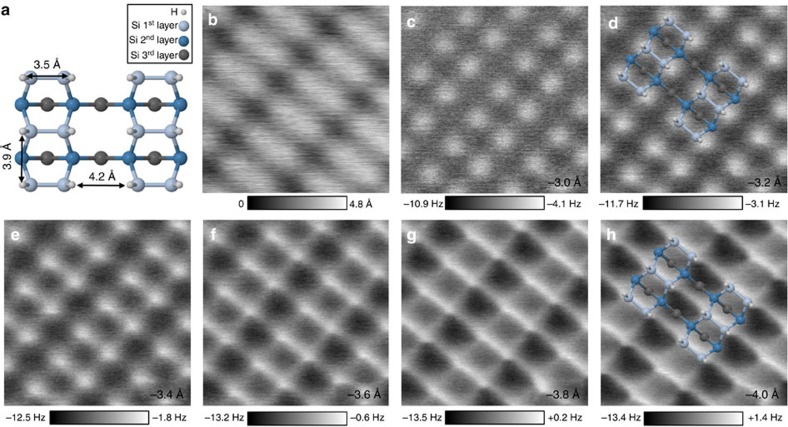
Series of frequency shift maps at different tip elevations. (**a**) Ball and stick model showing three silicon layers of the H–Si surface in the 2 × 1 reconstruction. (**b**) (2 × 2) nm^2^ constant current (30 pA, +1.2 V) STM image acquired with a passivated tip. (**c**–**h**) Series of raw NC-AFM frequency shift maps of H–Si surface at different tip elevations. Images are recorded at 0 V and with an oscillation amplitude of 1 Å.

Figure 2a presents a ball and stick model of the H–Si(100)-2 × 1 surface structure where we can notice the different *σ* bonds between silicon atoms, in particular the dimer bonds parallel to the surface plane and also the silicon back bonds. Using a stable passivated tip obtained following the method described in the previous section, a small defect-free area can be imaged in STM as in Fig. 2b. The feedback loop is then switched off, the bias set to 0 V and the scanner switched to AFM scanning mode. The tip position defined by the STM imaging set points before switching off the feedback loop is taken as a reference, that is, *Z*=0 Å. Figure 2c–h shows a series of AFM frequency shift maps at different elevations . Since these images are taken in constant height mode, more repulsive tip-sample interactions appear brighter. In this example, substantial contrast starts to be visible at *Z*=−3.0 Å (Fig. 2c) where we clearly see single atoms appearing as a bright protrusion and organized in a clear 2 × 1 reconstruction. As the tip is brought closer to the surface by 0.2 Å in image 2-c, the signal to noise is improved and the atomic contrast is clearer. Superimposing the surface model to the AFM image allows us to further highlight that, at this tip-sample distance, only the hydrogenated silicon atoms are visible.

However, as we keep decreasing the tip-sample distance, we start to see bright and sharp bond-like features appearing between atoms of a dimer as clearly seen in Fig. 2e at *Z*=−3.4 Å. These features appear to be due to the silicon dimer bonds. In addition, we notice features consistent with the back-bonds between dimer and second layer silicon atoms in accordance with the surface model in Fig. 2a. We note here that although this surface was previously investigated both experimentally^32^,^33^ and theoretically^34^,^35^ using NC-AFM, the evolution toward images consistent with the known bond structure as reported here is unprecedented.

Interestingly, when decreasing the tip elevation to *Z*=−3.6 Å in Fig. 2f, we see that in addition to the intra-silicon contrast enhancement, new sharp features appear in the inter-silicon dimer row region between two hydrogen atoms. These appear more pronounced in Fig. 2g,h. Unlike what appears to be the bond contrast corresponding to the silicon dimer bond, the feature in the inter-dimer region does not correspond to a real chemical bond as can be understood from the ball and stick model of the silicon surface (Fig. 2h). Moreover, the model shows that this AFM feature also does not correspond to the position of third-layer silicon atoms.

While the above associations of image features with known structure appear compelling, we must be cautious and acknowledge that tip and substrate geometries are substantially altered during imaging, especially at very small tip heights. To determine the unperturbed substrate structure, it is necessary to create a candidate structure and subject that to a simulated imaging process at a range of tip heights. Simulations done in this way capture force-induced alterations of structure and thereby result in modelled images that can be compared with experiment. We describe the modelling process and discuss the origin of image features in the following discussion.

### Reproducing AFM images using DFTB

To simulate AFM images, it is important to choose a correct level of theory to properly consider the necessary undergoing physics and chemistry while keeping the calculations tractable. In addition, the atomistic definition of tip and substrate is a requirement in many cases. Among first-principle frameworks, DFT is the first obvious choice, especially when dispersion correction has been considered to include the small long-range forces at large tip-sample separations. Unfortunately, DFT is computationally expensive for many systems, especially those where imaging must be done for a bulk structure, not only a molecule. Here we use DFTB, which at a lower computational cost can provide results comparable with DFT using traditional semi-local functionals for the silicon-based systems^36^.

**Figure 3:**
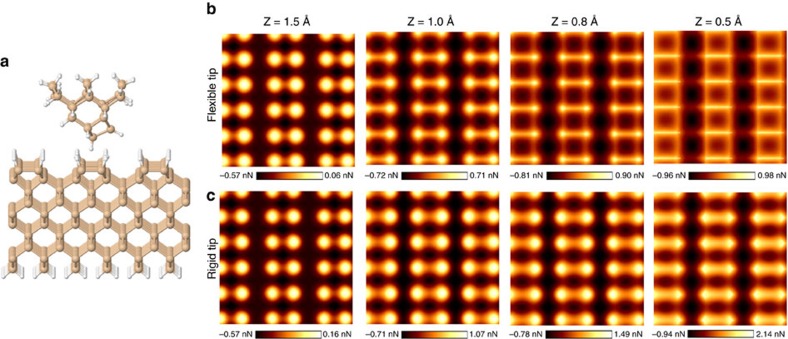
Simulated force maps from DFTB calculations. (**a**) Tip structure and H–Si slab considered in the DFTB calculations. (**b**,**c**) Series of simulated (2 × 2) nm^2^ force maps at different elevations using a rigid and a flexible tip, respectively. Force maps in **b** were converted to frequency shift maps and are shown in Supplementary Fig. 1

The modelled system is shown in Fig. 3a. The pyramid-like reconstructed structure considered for the tip ends with a tilted passivated silicon dimer so that the apex is a hydrogen atom. This tip consists of silicon and hydrogen atoms as an approximation to the passivated AFM tip used in this work. Similar model tips, called ‘dimer tip', have been previously studied in the literature and satisfactory results have been reported^13^,^37^,^38^. Here we placed more bulk structures at the base of the tip which, along with the hydrogen passivation of the silicon dangling bonds, result in higher stability. This structure can be geometrically optimized by various *ab initio* methods without the need to freeze the base atoms which leads to an unstrained structure increasing the fidelity of the forces read on the tip atoms.

For the substrate, a super-cell consisting of a H–Si(100)-2 × 1 silicon slab containing three dimer rows with six dimers per row is used. The slab consists of 10 silicon layers with the bottom one terminated with hydrogen atoms. The lowermost two silicon layers of the slab and the uppermost silicon atoms of the tip, along with their passivating hydrogens, are fixed to allow the constant height criteria of AFM. The rest of the atoms are relaxed to a force threshold of 0.02 eV/Å.

Initially, the tip has been placed at different elevations with respect to the substrate. The height is measured as the distance between the topmost substrate atom and the lowermost tip atom. The forces on the tip atoms are read after the relaxation, then the tip is shifted by 0.1 Å in *x-* or *y-*direction for the next point calculation. The scans at each tip elevation are performed from one hydrogen atom, to the next equivalent hydrogen atoms along and across the dimer rows. At each elevation, there were about 3,000 geometry optimization calculations with the results shown in Fig. 3b. An animation showing the tip and surface atoms relaxation during force reading is included (see Supplementary Movie 1). The scans are tiled for better illustration.

In good agreement with the experimental results, we see that at higher tip elevations, the dimer atoms appear as bright protrusions. As the tip approaches the surface, the atomic features start to dim while features in the silicon dimer bond region start to appear. Finally, at very low elevation (0.5 Å), an apparent dimer bond and its constituent atoms are indistinguishable. In addition, we notice the false bond feature in the inter-dimer region appearing at lower tip elevation images, similar to the experimental results.

We next address the effect of tip flexibility in the imaging of this surface and also in enhancing the AFM topographic feature registered between adjacent dimers in different dimer rows, where we know with certainty there is no hydrogen bond or covalent bond. Atomistic modelling can provide useful insights in this regard. In the simulations referred to above, tip flexibility played a significant role. We can resolve that role by restricting some structural relaxations. We perform additional sets of simulations by fixing all of the tip atoms while letting the surface atoms of the substrate relax as before. The results are shown in Fig. 3c where one sees a thicker feature in the dimer bond region and bright atomic protrusions even at low tip elevation, which is different from the experiment. This is due to the lower freedom of movement for the rigid tip which causes stronger forces to be read on it. As a result, the bond contrast is somewhat lowered. Nevertheless, we still see what appears to be a bond contrast where we know the Si–Si bond is located. This shows that although the tip flexibility is not necessary to observe a chemical bond-like contrast over the dimer, it certainly enhances such contrast. In addition, the tip flexibility makes the inter-dimer contrast more visible. This is reminiscent of the debate in the literature about the role of the CO molecule flexibility to account for contrast due to bonds within molecules and between molecules^9^,^10^,^16^,^17^,^22^.

## Discussion

**Figure 4:**
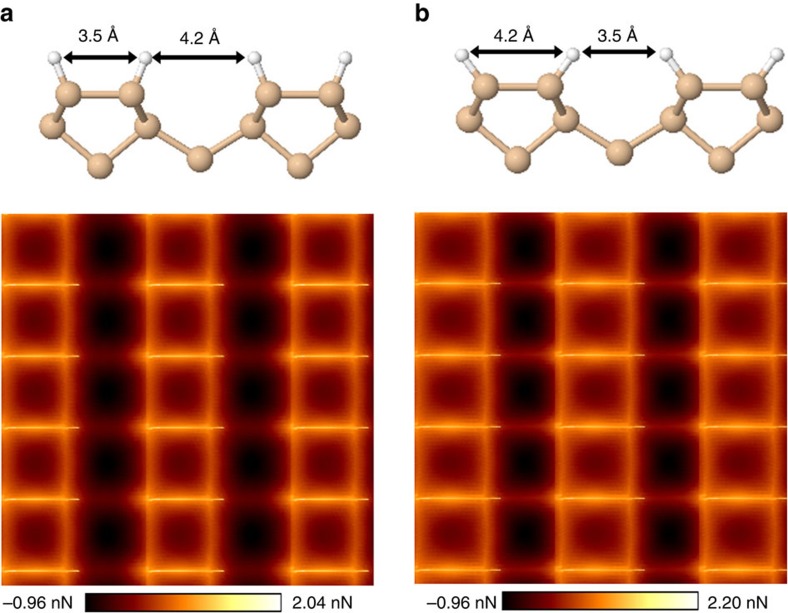
Simulated force maps for frozen slabs. Partial side view of the frozen slabs (upper panel) along with their simulated force maps (lower panel). In **a**, the dimer hydrogens are fixed in their relaxed positions, while in **b** they are slightly bent and fixed to obtain reverse distances between dimer and inter-dimer hydrogens with respect to **a**.

Are we seeing the silicon covalent bonds? To highlight the difference in the high-resolution AFM images between features corresponding to real chemical bonds and those appearing in the silicon inter-dimer region, we present additional calculation results using two different systems. The calculations are done at a very low tip elevation. In these cases the tip is flexible, but the substrate is frozen. In the first system, the substrate is as before with the atoms frozen at the relaxed positions. In the second system, the dimer hydrogens are bent slightly, while keeping their bond length at the equilibrium value (that is, 1.5 Å) so that the distances between dimer and inter-dimer hydrogens are reversed with respect to the equilibrium case, as shown in Fig. 4a,b. This gives us the opportunity to investigate whether the AFM chemical bond contrast is dominantly due to the Si–Si bonds, or it is more a consequence of the flexible tip scanning over closely spaced H atoms.

In the first system, what appears to be dimer bonds are visible as before, although some contrast is compromised due to the rigidity of the substrate. Interestingly, in the second system, the image contrast is still much sharper above the Si–Si dimer bond, despite the H–H distance being shorter in the inter-dimer region than over the dimer. If the feature seen in the dimer bond region were an artifact due to a convolution of a flexible tip with the H atoms attached to the dimer, we would have seen the dimer bond-like feature be diminished upon separating the H atoms as was done. Moreover, a stronger feature would be seen in the inter-row region than above the dimer, which is clearly not the case. This provides theoretical support to the idea that H–H orbital overlap is not the main contributor to the intra-dimer bond features seen in the experiment. While this result is consistent with the interpretation that the AFM is imaging the dimer bond, it is prudent to stop short of making that conclusion definitively as it remains conceivable that not all contributions to imaging have been accounted for. We note again at this point that, unlike other surface parallel bonded atoms imaged to date, these are *σ* bonded and not a *π* bonded atoms. Further and more detailed comparisons of simulated and observed images can build on the initial results presented here and could resolve the question more definitively in the future.

Furthermore, the results from Fig. 4b help explain the origin of the back-bond features seen in all of our experimental and theoretical AFM images at lower elevations. As shown in this figure, the distance between the intra-dimer hydrogens is 3.5 Å, which is less than the 3.9 Å between adjacent H atoms on different dimer rows. Yet, we still see much more prominent image features where the silicon back-bonds are expected (Fig. 2a). It appears, within the calculations, that the silicon atoms and possibly the Si–Si *σ* bonds are the major contributor for the bond feature contrast corresponding to the back-bonds, and that the H–H overlap plays a minor role here.

To summarize, we established that a hydrogen-passivated tip can be reliably prepared and identified. We use this passivated tip to image the H–Si(100)-2 × 1 surface. Using a DFTB-based approach to AFM simulation, we could successfully reproduce the evolution of AFM images at different tip elevations. We show that tip flexibility can enhance and sharpen the appearance in AFM images of what are known to be true covalent bonds. Moreover, we prove that non-bonded atoms in close proximity can appear bonded, and that false impression is enhanced by tip flexibility.

Because of the technological importance of the H–Si surface for prospective quantum silicon based^28^,^29^,^39^,^40^,^41^,^42^ and hybrid silicon-molecular technologies^43^,^44^,^45^, this work opens up the possibility to study building blocks for those technologies such as silicon dangling bonds and single molecules adsorbed on the H–Si surface using combined STM and NC-AFM.

## Methods

### Experimental techniques

Experiments were carried out using a customized commercial LT-STM/AFM system (Omicron) operating at 4.5 K and controlled by Nanonis electronics. We used highly arsenic-doped silicon (100) samples (Virginia Semiconductor, Inc.) with a resistivity of 3–4 mΩ cm (∼1.5 × 10^−19^ atom cm^−3^). The H–Si(100) samples were prepared in a ultrahigh vacuum chamber with a base pressure of 3 × 10^−11^ Torr. They were first degassed for about 12 h at ∼600 °C then flashed at high temperature (1,250 °C) before hydrogen termination. The followed sample preparation procedure, described in detail in ref. 46, ensured a high-quality H–Si(100) surface with low-defect density.

AFM data were acquired using a qPlus sensor equipped with a separate wire for tunnelling current^11^,^47^, showing a Q factor ∼10,000 and a resonance frequency of 26 kHz. Sharp tips were obtained by electrochemical DC etching in NaOH solution the 50-μm-thick polycrystalline tungsten wire glued to the sensor. The qPlus was cleaned in ultrahigh vacuum in a FIM attached to our LT-AFM system. It first underwent a series of ebeam heatings with the tip at 500 V and 1 mA for about 15 s, then was further cleaned by field evaporation in FIM^29^.

The AFM images presented in this paper were acquired in constant height mode, that is, frequency shift maps, with an oscillation amplitude of 1 Å. Care was taken to minimize drift during the image acquisition, typically of ∼20 min per image, by settling for about 12 h after the approach to allow piezo stabilization, and using an atom tracking module as implemented in the Nanonis controller. The sensor's oscillation amplitude was calibrated using the tunnel current method^48^. To avoid cross-talk problems and artifacts in frequency shift measurements, for example, phantom force, all AFM data were acquired at 0 V (refs 47, 49).

### Theoretical calculations

We have used DFTB method^50^ as implemented in the DFTB+ code^51^. To include dispersion interactions, a Lennard–Jones type pairwise potential based on the universal force field^52^ was used. The Slater–Koster parameters used here are based on ref. 36.

Atomistic structures were visualized by Jmol^53^. Simulated force maps were produced using Matplotlib^54^. Animation in Supplementary Materials was made using VMD^55^ software.

### Data availability

The data that support the findings of this study are available from the corresponding author upon request.

## Additional information

**How to cite this article:** Labidi, H. *et al*. Indications of chemical bond contrast in AFM images of a hydrogen-terminated silicon surface. *Nat. Commun.* **8,** 14222 doi: 10.1038/ncomms14222 (2017).

**Publisher's note**: Springer Nature remains neutral with regard to jurisdictional claims in published maps and institutional affiliations.

## Supplementary Material

Supplementary Information Supplementary Figure and Supplementary References

Supplementary Video 1 Supplementary Movie 1. Animation showing the tip and surface atoms' relaxation during DFTB calculations of a part of the image simulation at small tip-surface distance. The bending and rotation of bonds is visible giving a sense of the interactions and atomic relaxations involved. The animation was made using VMD software (Supplementary Reference 2).
