# Supplementary material for: Indications of chemical bond contrast in AFM images of a hydrogen-terminated silicon surface
Source: Nat Commun. 2017 Feb 13;8:14222. doi: 10.1038/ncomms14222 (PMC5316802; doi:10.1038/ncomms14222)
Supplement: Supplementary Information — Supplementary Figure and Supplementary References [file ncomms14222-s1.pdf]

Supplementary figure

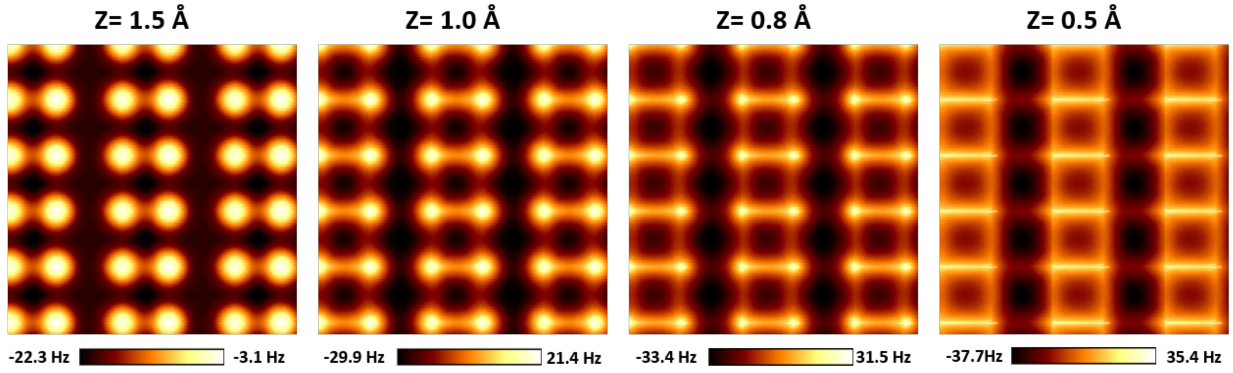

FIG. S1. Series of (2×2) nm^2 frequency shift maps at different tip elevations converted from the simulated force maps presented in Fig. 3-b of the manuscript (case of a flexible tip) using the conversion method introduced by Giessibl [1]. We used k= 1800 N/m and the same parameters as in the experiment: A_0= 1 Å, f_0= 26 kHz

Supplementary References

[1] F. J. Giessibl, "A direct method to calculate tip–sample forces from frequency shifts in frequency-modulation atomic force microscopy," Applied Physics Letters **78**, 123 (2001).

[2] William Humphrey, Andrew Dalke, and Klaus Schulten, "VMD – Visual Molecular Dynamics," Journal of Molecular Graphics **14**, 33–38 (1996).
